# Supplementary material for: The protein and volatile components of trail mucus in the Common Garden Snail, Cornu aspersum
Source: PLoS One. 2021 May 27;16(5):e0251565. doi: 10.1371/journal.pone.0251565 (PMC8158898; doi:10.1371/journal.pone.0251565)
Supplement: S2 File — (DOCX) [file pone.0251565.s002.docx]

**Contig 104 – Hemocyanin isoform 1 (0.0)**

MAPIMVWLWFTCMLVSSNALLVRKDVDHLTPEEVLNLQKALREVNKDTSSKGFAAIAAYHGYPPQCKHGSKAVACCVHGEPTFPQWHRLYVVQLEQALKEKGLNIGIPYWEWTHQLDHLPDLVSQRVFIETDGGKARSNVWYQGEIPTPEGVKKTARAVDPRLFQQVVAGQYTDLFEHVLNALEYPNYCQFEVQFEVAHNTIHYLVGGRHTYSVSHLEYTSYDPIFFLHHSNVDKIYTIYETIQRARGYTPGPGCTGECELCDIVGFRTPLEPFSRDSNPFALTKTHSHPYEATENTLFGYKYDNLTLNGLDVNKIKGIIEKRQAADRAFASFRLHGIGVSADVRVKVCVSDSHHLAADYCEFAGNFFILGGPLEMPWTFNRPYYFEITKTVEAMNISFTSDYHVEIDVYSVNGTQLPSDILPHPSVSFRPGKGKSDPEIVYELEHKEEVSIRKDVDHLTREEVLELREALEKFQSDHSVDGYQAIAEFHGDPGKCPFPTAKDRFACCIHGMPNFPHWHRLLVVQVEDALRRRGAHTGIPYWDWTKPNKIPVLAADETYINPHDNAEHVNPFHHAVIGFLGGDAKTSRDPLPELTQTPKWGDHTELFDVFLLALEQDNFCDFEVQFEIAHNLIHGYVGGNSKYGLSSLSYSAFDPIFYLHHSNIDRIWAIWTALQQQRGKPYKAHCAQSYVYTPLKPFAFHTPYNNNEKTFAHSTPTEVYNYEKEFQYGYENLEFGGFGIPELENYINEHLKSKPRTFVGVHLHGIKTSGLATIHVSASGKDYVAGHFAILGGPSEMDWEYDRPYRHDISHALEELGVNWAQPFDVTIEMHTFDNKPIDVSQFPRINIIHKEGEQLDETSQVTKITRKNVNHLTKEEVLNLRQALTFLEEDRSVGGYQTLGRFHGTPNWCPYPSAEKKVACCPHGGPTFPHWHRLLTVQAENALRKHGYKGALPYWDWTQPATSLPEIVVPETYIDPSNNVETHNPFHDAHIDDVNQNTVRSVRDDLYQQPAFGEYTDIAKQVLYALEQDNYCDFEVQYEIAHNFIHALVGGSEVYSMASLLYTAFDPLFYLHHSNTDRIWAIWQALQTYRGKAENAANCAIGKLRKPLPPFSLTSDVNPDPVTREYSSPLRVFNYKKSFQYEYDTLDFNGLGIPQLARVLEEHKSHDRVFAGFLLHGVGHSALVKFFICRNNTDCYNHGGEFYILGDSNEMEWSYDRLYKYEITEELQKLHLRYNDRYFIRYEIHDLTGEDLGQPFPAPTVIRQIGTSHLYGREYRDAVTAASHVRKDLDTLTAGEIESLRSAFLDIQQDHTYENIASYHGKPGLCQHEGRKVACCVHGMPTFPSWHRLYVEQVEEALLAHGSSVAVPYFNWITPLQKLPDLISKATYYNSREQRFDPNPFFSGKVAGEDAVTTRDPQPELFNNDYFYEQALYALEQYSFCDFEIQFEVLHNALHSWLGGHAKYSVSSLDYTAYDPVFFLHHANTDRLWAIWQEIQRYRGLPYNEADCAINLMRKPLQPFQDKTLNPRNITNIYSRPADTFDYRNNFHYEYDTLELNHLTVPQLESLLNRRQEYGRVFAGFLIHNIGLSADVTVYVCVPSGPKGKNDCNHKAGVFSVLGGELEMPFTFDRLYKLQITDTIKQLGLKVNNAASYQLKVEIKAANGTLLDPHILPDPSIIFVPGTRERQTEDGDVKHVVVRKNVDALSPRETLALIHALEALQADSSADGYQSIAAFHAVPPLCPSPSASKRYACCLHGMSTFPQWHRLYTVQVEDALRRHGSGVGIPYWDWTRASQSLPGFLSAENYTDPYTKEVHENPWHGASIDFEHSHTERDIQSAELFKLGPHGWDTWLFEQALLALEQEDYCDFEIQFEITHNAIHSWVGGSKEHSLAHLHYASYDPAFYIHHSNTDRLWAIWQALQKHRGYNPNEANCALEHIKDSLKPFSFGPPYNLNKLTTKYSHPEDTFAYEEHFEYQYDSLEFVGMTIPALDAYIKERQEHDRVFAGFLLRGLGTSATVDFTICDAFKKCYDGGHFTVLGGEQEKPWQFDRLYRYPITDVLKENHIHYDDDYHFHIHIKALNGTELDSKLIPEPSVLFVAAHEDVHVETTPNNIRHNLNSLEERDIQSLQAALRNLQRDTTNDGWASLASFHGAPARCPDPDHPKVACCVHGMPTFPHWHRLFALQIEQALHRHGSSIALPYWDWTLAIDELPSTFTKEDYYDVWRDEVVPNPFAHGYVASEDTYTVRDIQSPLHKKHDGKHSFLLHGVLDVLEQTDYCDFEVQFEVVHNAIHYLIGGHQTYSLSSLEYSAYDPIFFIHHSFTDKIWAVWQELQKRRNLPYNRADCALNYVNEPLKPFSFEGLNLNKFTREHAVPNTLFNHEDLGYAYDNFNIGGYDLGQLEKLIHDRQIKPRIFAGFLLKGIKTSGSINLKVCKLEECTSGGFFNLLGGPLEMPWAFDRLYKKDITWAIANIGLNPDDIHNTDSGFRLEVQAFNVEGTALPLSQVMPTPSIIYKPALGVDKDIHTTAVAGVGVRKDVTRLTVSETENLREALRRVKADNGSNGFQKIASFHGSPPGCVHENHSVACCIHGMANFPQWHRLYVKQWEDALTAQGAQIGIPYWDWTTAFTELPALVTEEDDNPFHHGKIYNGQNTTRAPRGRLFNHPEFGRESFFYRQILLAFEQTDYCDFEVQYEISHNAIHSWTGGQSPYSMSTLEYTAYDPLFLLHHSNVDRQFAIWQALQKFRGLPYNSANCAIQLLHQPMRPFSDSDNVNPVTRTNSRARDVFNYDRLNYQYDDLNFHGLSISELNDVLERRKEKARIFAEFLLHGLGASADITFDLCDSHNHCEFAGTFAVLGGPLEHPWAFDRLFKYDVTDVFSKLHLRPDSEFHFNIHIVAVNGTELDSHLIRSPTVQFVPGVKDYYEKIAQKIEIHDDVLLRKNINELSLEESANLRSALNKLQQDQGPNGFEAIAGFHGAPFKCPETGTDKYACCVHGMPVFPHWHRLLTVQFEQALKYHGAKEGVPYWDWTAPIGKIPSLFGDSADYNPFYSYTISFNNQRTTRDIQGELYNPHTINGFNYIYYLALSTLEEDNFCDFEVQYEVLHNEIHALIGGNGTYSMATLDFSAFDPFFMIHHSSLDRIWIIWQELQKLRHKPFNYAQCGGHVLDDPLHPFSFGDINKNDLTRLNSLPSSVFDYTHFGYEFDKLELNGHDVQGIDNVIHNLRHGNRVYLGFVLFGQQSSLEYKIDLIDDEGQAHTAGSFHLLGGEREMPWAYERLFKYDVTDVAKKFGITTDHPIKVKVTSTYYNGEPHQEYTDEIVAVERHADTDYDILVIPVSKDNTLVPKIVVKKGTRIEFVTSDLTDPLEDLGSYTTMHKCKIPPFSYNSYAFNTVHKLSPGDYFFVPKNAELCNAGRRIQITVEDD

Contig 1055 – Protocadherin Fat 4 (0.0)

MSMPESMIFLLQLLHLMLIGTHLCTPLILPTXXXXXXXXXXXXXSSTTNVFTFTVIVSDQGTPTAKTCSTIIRFTVSTDLAPYFTPATYTWSAVPENAFELQSTFTVIGRDDDRQGNLQYKVIGLWAGPEYFSIRRTNPQQPDSTGTVFVNRTLMSDNDLTYTVIAVVYDDARPLLSGTATITVNLIRNPSGPIFSLRDYNATFNELEPIGYNPVNVSAVDPDGDIVTYSLLTTNVDQKALQYFDIIPTSGKIIVKQSLTMDQAKQTTPFVLTALARDNRQPQKTDMATVRLYVTRNLYSPRFINTPYNVPAISENQPINSVIYATVSAVDQDATGPLVYEEIANTTAAYYFNIDRSTAAVTLANSLIAGTSDVYQLYVRTYDPSYPADYAEEVVNIRVNRNEYGPVFNQSAYVITINETESIGTNILTVSATDNNTRDVVTYNATGEQDTLTLFQIFSSGQILVKQSLLGVPKDTYTMNVLARDNGTPSRSTPVSVTINILRRPGNPTLGPAVCQDTISENRNPGPINVRITASDPNAMGPLMFEEIGEYPAPTFFSVNNSGVIFLTQNLRNNSVRDSSVVYVVRVYDSLRPDRSSQIRCTITILHNQNPPRFTLPNYTVTIRDTHPLLAFVTNVTAIDIDPQDVVTYSIVGESIQRDLNTGMSDYFLIDTTNGTFYLRKSVTGSGIRQFVLRVRACDNGYPQLCAEADVTVIVDSSGVAPTMSNRQAQINENAQRGDFVLQLTATDADMAATSFIVYEFVNPPPAYFQLDARSGNITVAKSVFYDNQTVYMFDVQAYDQADPSRKARATVTINVVRNQYGPVCQMSISRFTISEYFPVPSLIATIVATDTDRDPLLYTLISTMPAPANASDPTFYVDSRNGNLWLFHSQEGTQVTTYNLTIRVSDQRGASEKRDNCGVLITIDLDITPIFTNGPADRTVGEFASPGPVSLVQATDGDLEGQIVYQLTGVYPSPSFFSVNNASGQINLISSLVNDPLNLTSYTLTIIAFDSAHPKRTASTTVIITVPRNPSAPTFPQSSYSVIIPETTALGSVVIALNATDADNDTLTYSITNSMPANGTNFFFMDGNLLKTRDNLRVAADNYMLTATARDPRGKTGNALVGISITRVVSDRPPQFTMQTYTTVITSYQAVGSPVLPTTAIDPDIPNNPERIKYRMTAPSNFSFFNIDQNTGVISVSRPLSQDSTRASYYTMELYAYDEQNPSQVATAFAVIYVDHNPGTPVMAVPSYTATISEYFPLGVSVIKVTATDSDNNTVRYYIQPDSAGIVASEFFSISPNDGIIRVIKPLTNNTQDQNTFSVVAYDDGIPSKSVVGTVTIRILRDRFAPTCASRSPVSVPENAFVNSTTPLFTFVATDADNSGNPIIYGPSGEGIGRVFFHINTNGDVFVSFPLKETSVQSFNLTAQVYDQSNPQKLGYCTATIVVVRNPSSPQFNASSIVRSVYEYELSGYIIADLDAVDADGDTLRYYISGDAESLSFFDVDDQSGLLTVKRPLSETARNQFVFSVLASDRRTPEHTGAMSVTINTVRDQRPRFVSVPTSVDLSETDKRGKIVTNVTAVDSDLRGQIRYAANSTDGSEAFFSIDPVTGFISIIRDLNTDISTSYEFVIYAYDSLLPQVHSLPAVIRFNVARNPSGPVFQNVPYRFALDVTSSGDRLGTRIGCVNATDLDGDIPTYRISSTDNAANYFFIDGSSGCIYLVNSLTGLPPSQTQLQFSVIATDRPQLSRTAMENVIITLSRDDSLPMFSQGSYTVTINETQSVNSIILPGIRAVDSNLQGMINYDVTGIYPADLFFDIGTTDGNLRVISDLRTDSQPRPQYLVKVIAYDTA

**Contig 35651 – Hedgehog intein domain containing protein (2.76e-36)**

MVSMKLAVLLAVAAVVALSSSAVVDPTVGGNVSENLIHDNKVKDDTPIGSDLTEDNDTNDGKVDKYAKIDGIALNSLTEDELKDLLDDEDNIVAAELDIIVGEKDVALRKNEAAYEISNGKPQRRQRRWIPVALRLFSAGRALFRGAARAPKPTVSGSRVTQSYTRPGSYNDAVRDFNRFRPDNVRSFNKNGISGQTGTVGNHRFTVRDGSKQGSPTLEIRSPKPNGEHVRKFRYNQK

**Contig 27195 – Cadherin 99C isoform X2 (5.95e-53)**

MDLKNQIVYVLAVASLAVSVMAQKKACEPLDDGPFLATLPTLNFMIPEASDTDLQDAERTGSKKYFSELRIFGDPTADFNLTVFANILDPFEYFTVVTEAGTNYLKLIKPLDRDGSTSSPEDDVDFIPFTLTCQPQNASLSEKIYDARIFITDVNDNIPIFPKGNNTLNFTLSEATPAGTTLFTAEADDKDAHLDGNITYALNNYANLFSVDNFTGAVTLISELDYESLPPGKTIALILTAQDSELGVTYTVTATATIHVTDADDQGPAFIYQGCFTYMSVCAWPKYTTGLSLKKDQPIFQPVPNKGQGPV

**Contig 50074 – Phospholipase B1 membrane-associated like *Biomphalaria glabrata* (1.17e-148)**

MRMGHLGISLCLLVCLCMSAPAGAKGRGDQEAEILRQLLLRQHVNRTELDREYIATTQKRSYTFHCPVLPPSPSTPVSVHRLRPGDISVVAALGDSLTAGRGVELGILGLLIDFPYLSWSIGGDGSYSSLVTIPNILRNYNPYLYGYSVKAGLNSRRFNVAETGANSRDLLAQAEKLVQLMRDDTNVDFSHSWKLITIFIGANDLCDYCQDEDAYTAQAYFNNLKTTLDYLQAHVPRAFVNLVEVLNVEMVRSLGENPLCRAVHNAVCKCVANPANEEEARRAVQLREEYQNKTRDLVASGRYDTQENFTTVLQPFYENTTLPATPSGSVDLSYFAKDCFHYSRKGQQTVAEALWNNMFEPVGSKDNSWRPGQPIKCPTEESPFLYTAINSNRAGT

**Contig 303903 – Collagen alpha 1 chain isoform X2 (223e-168)**

MFSFVDLRLLLLLGATALLTHGQEDIPEVSCIHNGLRVPNGETWKPEVCLICICHNGTAVCDDVQCNEELDCPNPQRREGECCAFCPEEYVSPNSEDVGVEGPKGDPGPQGPRGPVGPPGRDGIPGQPGLPGPPGPPGPPGPPGLGGNFASQMSYGYDEKSAGVSVPGPMGPSGPRGLPGPPGAPGPQGFQGPPGEPGPGGSGPMGPRGPPGPPGKNGDDGEAGKPGRPGERGPPGPQGARGLPGTGLPGMKGHRGFSGLDGAKG

**Contig 3063 - Collagen alpha 1 chain (3.92e-134)**

MATYIVRKVVPANPVSFCVIRSFLFSSAKPKTSLPCSGSVLCLRSFLSTTKRFSSHVSEEKPKKPHCNVGTIGHVDHGKTTLTAAITKVLSEVYSSDNKFVSYEAIDRAPDEIKRGITINAAHIEYVTPYRHYAHTDCPGHIDYVKNMITGTSQMDGAILVVAATDGTMPQTREHL

**Contig 314246 – Collagen alpha chain like (5.89e-17)**

MKMIVLAGLCLLATASAYILTDEIQQPAPQCTTDSKADLFVMIDESSSIAKIGFSRLKVYXLRNLIFGPDYIRVSIALFATKYRILCTFNSYTTVDEMVALIDSIKQEGGWTYTDLAMSELIASGVLTDAKYGRRPGVPAVYLMITDGNSRNRTATCEESDQIGKLGFIRKVITVGSVDKDEALCIAGGDDKSVFNVRSYNALADLTIEIVTAICTVPPPPPATPTCPNPQKIDV

**Contig 3727 – Collagen alpha chain like *Biomphalaria glabrata* (0.0)**

MRHRQMGNGGSWFASVLLLLGLFDTTHAAECLGCAPPCICEGQKGFPGAPGLPGLRGPEGLPGFPGNEGPPGAAGLPGDQGEFGAQGEKGFRGQEGLAGFTGSPGIPGQPGLEGPVGPEGYPGCNGSKGERGPAGQPGPKGYTGSSGFPGFPGMKGEPGEFLGVGIGFKGDPGEQGKPGLQGSRGLPGEEGDVGPIGDPGIPGRRGIDGLIGEKGEKGNDVFGKEGPPGDKGPQGDDGVAGVVGSSLNQTIVIGAKGESGDKGIPGIKGDVGSRGYEGIPGAQGVPGVKGPPGMEGPAGRRGKRGKDGPPGDFGPKGMVGEKGYPGISASDGSKGMTGDEGFPGIRGPGPPGRSGDYAEGVKGAPGPRGRDGEIGDSGFPGRRGNPGELGPPGITGPEGLPGFPGRAGVPGEPGISIKGDRGDPGVDGATGRDGAAGFPGLPGDIGYPGAPGANVRGPAGERGIPGVDGLPGQVGQPGEPGRRGEKGQPGIGPDIIGPKGLPGPAGDVGDTGFPGTSGLPGLDGEVGPRGEDCGSCPDGEPGIQGEQGDPGYGGANGRPGPDGFPGLKGEPGEPGLAGPRGPTGPPGFRGLDGDFGNGPKGQRGDNLYVEDVERFRGPPGPPGSIGYPGSPGRDGPMGDVGPRGLIGDMRHRQMGNGGSWFASVLLLLGLFDTTHAAECLGCAPPCICEGQKGFPGAPGLPGLRGPEGLPGFPGNEGPPGAAGLPGDQGEFGAQGEKGFRGQEGLAGFTGSPGIPGQPGLEGPVGPEGYPGCNGSKGERGPAGQPGPKGYTGSSGFPGFPGMKGEPGEFLGVGIGFKGDPGEQGKPGLQGSRGLPGEEGDVGPIGDPGIPGRRGIDGLIGEKGEKGNDVFGKEGPPGDKGPQGDDGVAGVVGSSLNQTIVIGAKGESGDKGIPGIKGDVGSRGYEGIPGAQGVPGVKGPPGMEGPAGRRGKRGKDGPPGDFGPKGMVGEKGYPGISASDGSKGMTGDEGFPGIRGPGPPGRSGDYAEGVKGAPGPRGRDGEIGDSGFPGRRGNPGELGPPGITGPEGLPGFPGRAGVPGEPGISIKGDRGDPGVDGATGRDGAAGFPGLPGDIGYPGAPGANVRGPAGERGIPGVDGLPGQVGQPGEPGRRGEKGQPGIGPDIIGPKGLPGPAGDVGDTGFPGTSGLPGLDGEVGPRGEDCGSCPDGEPGIQGEQGDPGYGGANGRPGPDGFPGLKGEPGEPGLAGPRGPTGPPGFRGLDGDFGNGPKGQRGDNLYVEDVERFRGPPGPPGSIGYPGSPGRDGPMGDVGPRGLIGDMRHRQMGNGGSWFASVLLLLGLFDTTHAAECLGCAPPCICEGQKGFPGAPGLPGLRGPEGLPGFPGNEGPPGAAGLPGDQGEFGAQGEKGFRGQEGLAGFTGSPGIPGQPGLEGPVGPEGYPGCNGSKGERGPAGQPGPKGYTGSSGFPGFPGMKGEPGEFLGVGIGFKGDPGEQGKPGLQGSRGLPGEEGDVGPIGDPGIPGRRGIDGLIGEKGEKGNDVFGKEGPPGDKGPQGDDGVAGVVGSSLNQTIVIGAKGESGDKGIPGIKGDVGSRGYEGIPGAQGVPGVKGPPGMEGPAGRRGKRGKDGPPGDFGPKGMVGEKGYPGISASDGSKGMTGDEGFPGIRGPQGPPGRSGDYAEGVKGAPGPRGRDGEIGDSGFPGRRGNPGELGPPGITGPEGLPGFPGRAGVPGEPGISIKGDRGDPGVDGATGRDGAAGFPGLPGDIGYPGAPGANVRGPAGERGIPGVDGLPGQVGQPGEPGRRGEKGQPGIGPDIIGPK

**Contig 294250 – Peptidnickel transport system substrate binding protein *Bradyrhizobium* (0.0)**

MSFKLAACIMALGVAAIAASPASAQKKGGTLRLYHNDNPPSTSLLEESTIASVLPFAAVFNNLVVFDPAKVHESIDTVIPDLAESWSWDQTNTKLTFKLRQGVKWHDGQPFTAKDVQCTWRMLTGKSETQDFKRNPRKVWYSKLRDVNGEDEATFELTEPQPGLLALLASAFSVVYPCHVPQQVMRTKPVGTGPFRFVEFRRGDSIRLVRNPDYFKKDRPYLDEITVRSIDSRATRMLAFATGDYDITFPSDVSIPLMKDVKARAPNAICEMTSTNLQINLLVNRVNPPFDDPEIRKAMSLALDRKAFNSILFEGSGRLGGAMQAKPEGEWGMPQEILSTLMGYGPDTEKNLADAQVIMQKLGYSDAKPLSIKIQTRNLPTYRDPAVILADQLKKIYIVAELDILDTPRWYSRLQRKDYTIGLNVTGVSVDDPDGNLVENYSCNSERNYTQYCNAEVDKLLAAQSREVDKDKRRKIVFDIEHLLVDDAARPVILHSSAGNCWQPYVKNFHPHDNSQYNNLRFEDVWLDK

**Contig 2548 – Epiphragmin *Cernuella virgata* – (4.04e-27)**

MATQAWILLCAVLISTLPESHGLEFSINRTPGQGYCAHLRCLNDIDKKVSSFSTTRVTVYEVSSGGTRKVLASASADKPDVYIDPVRKDDVKGTGSFSQYHSEVSLDFNKEPDCKSGTFQCELEMLSQAGSKNVVRETVSPGYSEGECTCASITQKFTWLYDSANDAEKQLAVLKQSSSDISNAITSARQELNAKEKDIVRLRRNSTSLQGAINVLRQNNKDTEAELSVLRRSNEAMKDTVAQVQRNDDSQDEAIAELQHKDEAQKAVISTMQDNDKKINSKISELQGKDVAFAQSTDVFKKNDDDLQAAMSQLKSTSNVLQAIINDLKRNDSSIDVDIATLVHNDDVQAEDIANFQHHDQVHEQEIADHKQKDIILQEIIDNLLYNQTVISDALVNFQQRSAVVQQAISSLTQHDTETDAALAAHSQKDNVVSQLITGLKYNDTIFQQELDQEKSYDQVLFATISQLNKADDVLNAQANVLLENDVEFNARINVEIQSSSVLSRQLVLLTEQNTVLETSFVKLQETFEQLRALFSKTRVTSITKTKTEISTSSESASSSVRTSSKSSYVSRKE

**Contig 19345 – Signal peptidase-like *Biomphalaria glabrata* (2.02e-155)**

MSTAGFVLTTLCVLASNLPVFQAVSEYAILEARPAGAKGEESKLSECVVFNNQHQSLPTVVSKNHFHRLVNLSHLQGCTPKDFIDSPVSLENSVVAVARGNCTFIQKASVVQSFNASAILVVDYPNTTIPTYPGGNESDFKSLTIILATIASADFKEVLRLSKSVDMTLYVPANPLWDANMILIIVLSTILVMSGAAWSAYDVWQFPKRSRRKSLKSRDSEDNEEEGGAQKIENEITVFTIMIWFVLICAMILLLYFFYDYMVYFFIAVFCISGTYSLYHCLLPLWSRCLPVTYDIPVDKLPCINSKVKLRSFLLLLLCATLGIFWAIQRHSRYAWVLQDLLGAAFCVFFMHTLRLPNLKVIAMLLILLLVYDVFFVFITPHFTNDGNSIMESVATGGKGHSKESLPMVFLLPTLSDFPLRHCMDREFSLLGFGDVIIPGLLVSYNAXXXXXXXXXXXXXXXXXXXXXXXXFPHLYELWTASPLILGAWDSLHYGCGGPYT
