# Supplementary material for: The protein and volatile components of trail mucus in the Common Garden Snail, Cornu aspersum
Source: PLoS One. 2021 May 27;16(5):e0251565. doi: 10.1371/journal.pone.0251565 (PMC8158898; doi:10.1371/journal.pone.0251565)
Supplement: Attachment — Submitted filename: Response to Reviewers for Manuscript PONE_2_SC.docx [file pone.0251565.s003.docx]

**Response to Reviewers for Manuscript PONE-D-20-38531**

*Editors Comments*

1. Style requirements met
2. Reference list correct
3. Genbank accession numbers have been uploaded to NCBI and noted in manuscript
4. Added to cover letter
5. Added to cover letter

***Reviewer 1***

The manuscript is very well written. The rationale presented in the introduction is convincing, noting that I am not well versed in the chemical ecology of gastropods. I was left wondering if non-protein components, like those synthesized in mucous-producing cells, may be important for contact sensation via the mucous. I realize that this may be beyond the scope of this study, but it may be worth mentioning in the introduction or discussion.

Response: Added to discussion (Line 514).

Do we know the active ingredient in On-guard Snail Gel? The methods section looks very complete. I have no concerns here.

Response: In spite of examination of the label and an email to manufacturer (in UK), the active component has not been confirmed. As the product is stated as being non-toxic and natural, it is likely that it contains a combination of natural ingredients that the snails find aversive.

I think it would be helpful to readers to use the full name of each tissue on the bottom axis of the heatmap in Figure 3 for quicker reference. It looks like you should be able to fit them into the space available, perhaps at an angle. Likewise, you could insert the corresponding chemicals in Figure 5 at each peak.

Response: Chemical names have been included within the chromatogram to ensure easy reference while maintaining tidiness and clarity.

It could increase visual interest to include one diagrammatic image or labelled photograph of the relevant snail anatomy. Though very basic, this could help gastropod novices become more connected to the work. This could even be a figure inset.

Response: To help readers comprehend the anatomy, we have added an anatomical figure (as 3A), inclusive of abbreviations used in heatmaps 3B and 3C.

I only noticed one typographical error: Line 108 –punctuation error.

Response: Corrected.

***Reviewer 2***

When the authors measured the effect on the heart rate of contact with mucus, it would be interesting to look at one or more mucus samples from distantly related organisms, rather than just water as a negative control. Are the snails detecting common mucus molecules, or something unique to that species?

Response: Agreed, and are indeed exploring this type of analysis between related species within the scope of another research project performed by the first author, i.e profiling mucus molecules between sympatric pest land snail species.

What were criteria for selecting the proteins in Fig. 2? The authors choose to present 10 of the 22 proteins that were predicted to be secreted. Why were these 10 selected and not the other 12? Were they more abundant, or was the selection arbitrary? Notably, two of the contigs that had a markedly higher relative expression in foot (104, 2548) are not included in this ten; why not?

Response: The 10 that are presented were thought be the most likely to play a role in communication and are generally of more interest. Eg. Novel or uncharacterised. The remaining 12 contain sequences that match to several variants of collagen and another variant of epiphragmin. These sequences are available in the supplementary material (S2).

In figure 3, the authors present relative expression compared to other tissues. It would also be interesting to provide the TPM values to give a sense of which were the most highly expressed proteins in the mucus.

Response: This is the purpose of heatmap Fig 3C, which has now been more clearly explained in the figure legend.

Lines 160-162 state, “A transcriptome-derived protein database was prepared by combining the foot and anterior tentacle with tissue transcriptomes of the mucous gland, central nervous system (CNS) and posterior tentacle using the CLC genomics Workbench.” It is not clear at this point where the non-foot transcriptome data comes from. Later in the paper it is identified, but it should be made clear here. Additionally, the methods only state that foot tissue was used, but later in the methods it is stated that anterior tentacle was also used. This should be cleared up.

Response: Apologies for the oversight – the anterior tentacle was removed and RNA isolated at the same time and using the same methodology as the foot tissue. This has been added to the methods. (Line 152).

Line 172 states, “Trail mucus biomolecules were isolated using Sep-Pak plus C18 cartridges (Waters) according to the manufacturer’s instructions.” Give more information on this separation. Was this FPLC? What was the buffer system? Liquid chromatography of snail mucus is challenging due to the large glycosaminoglycans, which have a tendency to clog columns.

Response: Apologies for the oversight – The use of 100% acetonitrile for cartridge preparation and 60% acetonitrile for elution has been added to methods (Line 176). This pre-preparation, prior to LC-MS, is an approach that would remove large glycosaminoglycans.

For the second extraction method, were all visible bands analyzed by LC-MS/MS? If not, what were the criteria for inclusion?

Response: Yes, all visible bands were subjected to in-gel trypsin digestion and analysis and this information has now been added to methods (Line 184 – ‘All visible bands were excised….).

Were there notable differences in the proteomes derived from the two extraction methods?

Response: Yes, and this information has now been added to the manuscript (Line 396).

Note that C-lectins with similarity to perlucin have also been identified as centrally important in the adhesive properties of a terrestrial slug’s defensive mucus (Smith et al., 2017, “RNA-seq reveals a central role for lectin, C1q and von Willebrand Factor A domains in the defensive glue of a terrestrial slug” Biofouling).

Response: Thank you for drawing our attention to this very interesting paper. A reference to this paper has been added to our manuscript (Line 348).

Fig 3. Provide some guidance for interpreting Z-score in the figure legend. I assume that red indicates higher relative expression. Not everyone will be familiar with Z-score.

Response: Apologies for the confusion. Yes, red indicates higher expression and this information has now been added to the Figure 3 legend.

The last page and a half of the discussion could be more focused. There is some repetition that could be removed.

Response: Agreed. This part of the discussion has been revised and we now feel it is more focused.
